# Risk assessment tools for QT prolonging pharmacotherapy in older adults: a systematic review

**DOI:** 10.1007/s00228-022-03285-3

**Published:** 2022-02-14

**Authors:** Simone Skullbacka, Marja Airaksinen, Juha Puustinen, Terhi Toivo

**Affiliations:** 1 Clinical Pharmacy Group, Division of Pharmacology and Pharmacotherapy, Faculty of Pharmacy, University of Helsinki, Viikinkaari 5 E, P.O. Box 56, 00014 Helsinki Helsinki, Finland; 2 Unit of Neurology, Satasairaala Central Hospital, Satakunta Hospital District, Pori, Finland; 3 Clinical Pharmacy Group, Division of Pharmacology and Pharmacotherapy, Faculty of Pharmacy, University of Helsinki, Viikinkaari 5 E, P.O. Box 56, 00014 Helsinki, Finland; 4 Hospital Pharmacy, Tampere University Hospital, Pirkanmaa Hospital District, Tampere, Finland

**Keywords:** Risk assessment tools, QT prolongation, Torsades de pointes, Older adults, Risk management

## Abstract

**Purpose:**

Many drugs are associated with the risk of QT prolongation and torsades de pointes (TdP), and different risk assessment tools (RATs) are developed to help clinicians to manage related risk. The aim of this systematic review was to summarize the evidence of different RATs for QT prolonging pharmacotherapy.

**Methods:**

A systematic review was conducted using PubMed and Scopus databases. Studies concerning risk assessment tools for QT prolonging pharmacotherapy, including older adults, were included. Screening and selection of the studies, data extraction, and risk of bias assessment were undertaken.

**Results:**

A total of 21 studies were included, involving different risk assessment tools. Most commonly used tools were risk scores (*n* = 9), computerized physician order entry systems (*n* = 3), and clinical decision support systems (*n* = 6). The tools were developed mainly for physicians and pharmacists. Risk scores included a high number of risk factors, both pharmacological and non-pharmacological, for QT prolongation and TdP. The inclusion of patients’ risk factors in computerized physician order entry and clinical decision support systems varied.

**Conclusion:**

Most of the risk assessment tools for QT prolonging pharmacotherapy give a comprehensive overview of patient-specific risks of QT prolongation and TdP and reduce modifiable risk factors and actual events. The risk assessment tools could be better adapted to different health information systems to help in clinical decision-making. Further studies on clinical validation of risk assessment tools with randomized controlled trials are needed.

**Supplementary Information:**

The online version contains supplementary material available at 10.1007/s00228-022-03285-3.

## Introduction

Many drugs are associated with the risk of QT prolongation and torsades de pointes (TdP) [1]. TdP is a rare polymorphic ventricular tachycardia that can cause reversible syncope, ventricular fibrillation, and death [2, 3]. According to the QTDrugs Lists of CredibleMeds, about 200 drugs are associated with the risk of QT prolongation and/or cause TdP [1]. Based on ongoing and systematic analysis of available evidence, drugs may be placed into four QTDrugs Lists on the CredibleMeds website [1]. List 1 contains drugs that prolong the QT interval and are known for their risk of TdP, even when taken as recommended [1].

Several risk factors for QT prolongation and TdP are known [3, 4]. QT prolonging drugs were responsible for 48% of the cases of QT prolongation in a Canadian study [5]. Several non-pharmacological factors, including female sex and age ≥ 65 years, several diseases, as well as electrolyte disturbances were found as risk factors in a quite recent systematic review [4]. Patients with multiple clinically recognizable risk factors, like older adults with polypharmacy and comorbidities, have an increased risk for TdP [6, 7].

Concomitant use of QT prolonging drugs is common and has increased in in- and outpatients [8–10]. When contemplating pharmacotherapy with a QT prolonging drug, patient-specific risk factors need to be considered [6, 11]. However, the ECG screening rate of emergency department patients receiving QT prolonging drugs is considered low (20.9%) [9]. The American Heart Association and the American College of Cardiology Foundation have released recommendations on the prevention and management of QT prolonging pharmacotherapy and drug-induced TdP, mainly concerning ECG monitoring in an inpatient setting rather than other strategies that may identify patients at higher risk [6, 12]. The QT interval corrected for heart rate (QTc) is an indicator of TdP risk, but it does not fully account for all the risk by itself [6, 11, 13]. Use of a risk assessment tool (RAT) may be a more effective way to identify patients at risk of QT prolongation and TdP for whom repetitive or continuous ECG monitoring, discontinuation of QT prolonging drugs, or serum electrolyte concentration monitoring may be necessary [12, 14].

The aim of this review was to systematically summarize the evidence of different RATs for QT prolonging pharmacotherapy.

## Material and methods

### Data sources and retrieval of material

This systematic review was conducted according to the PRISMA guidelines [15, 16]. PubMed and Scopus databases were searched for potential studies from 2005 to 2017 (Supplementary Table S1). Author SS worked in a research team helping in the search process (author TT and an information specialist at the Medical Library, University of Helsinki). In PubMed, MeSH (Medical Subject Headings) terms were used and all fields were searched. In Scopus, the fields’ title, abstract, and keywords were searched. To avoid searching for duplicates in Scopus, the search was filtered with “AND NOT INDEX (MEDLINE).” The literature search began on September 22, 2017. Alerts on new articles from the databases were sent to the author’s (SS) e-mail. The search was updated in August 9, 2021.

### Inclusion and exclusion criteria

Any study design, excluding narrative reviews, was included. The RAT for QT prolonging pharmacotherapy could be used by any healthcare professional, in any healthcare settings. The RAT could involve anything from a risk score calculator to using computer software or databases to assess the risk. Studies involving risk assessment without using a tool or studies only involving ECG measurement and QT correction were excluded. The studies were included if they involved older adults (≥ 65-year-olds [17]) or the mean/median age of the study participants was ≥ 65 years. Articles were included if written in English and if full-text was available through the University of Helsinki Library.

### Selection of studies

Titles and abstracts of studies were read by author SS/TT. In cases of unclear articles, other authors also read the articles. Reference lists of included studies were reviewed. Studies were selected based on title and the article abstract. Full texts were retrieved for selected studies.

### Quality assessment of included studies

The included studies were assessed for strengths and limitations by considering the Grading of Recommendations Assessment, Development, and Evaluation (GRADE) approach [18]. The study design was considered for each included study and authors estimated the evidence level and risk of bias, if possible.

### Data extraction and analysis

Full-text articles were read (SS/TT), and the following data were extracted and analyzed qualitatively: country; study design, and setting; cohort or sample; number of patients; definition of long QT interval; QT correction formula used; description of the RAT used, strengths and limitations of the RAT, and the study and the primary user of the RAT. The studies were categorized and analyzed according to the RAT used. A detailed summary table of the included studies was made (Supplementary Table S2).

## Results

### Study selection and description of included studies

**Fig. 1:**
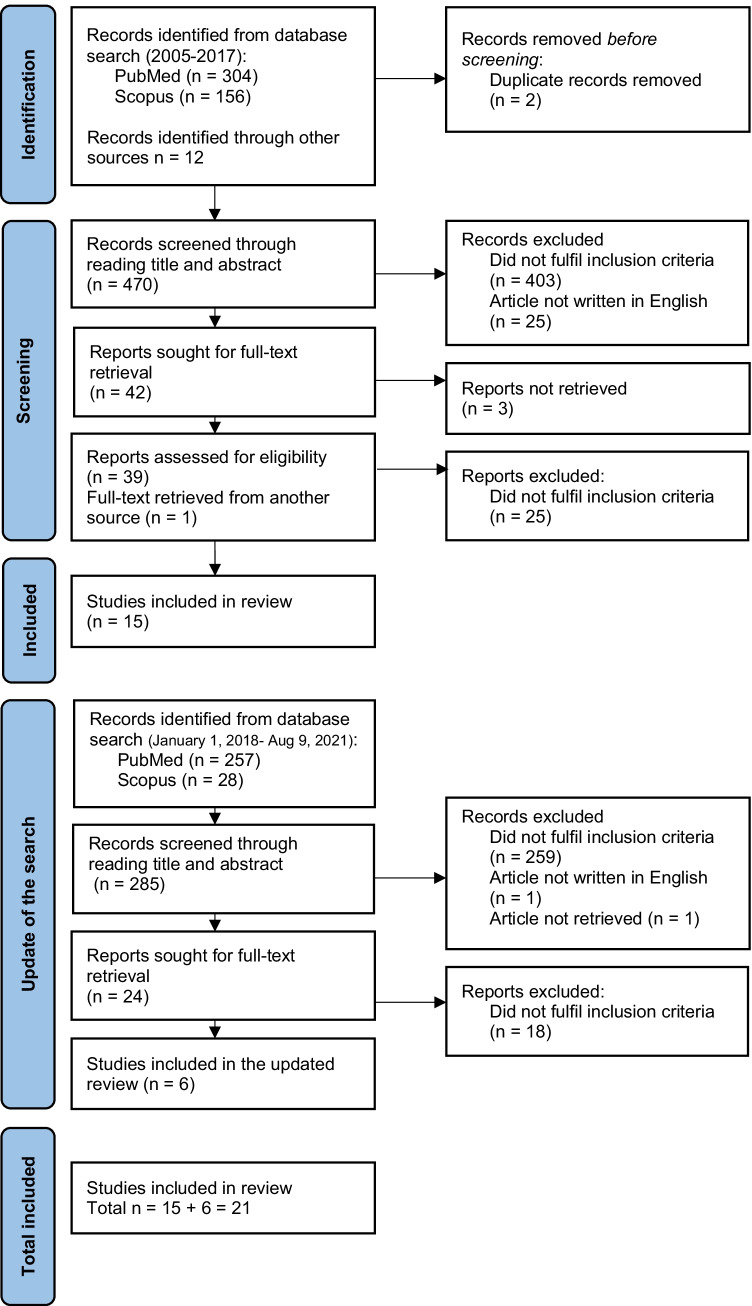
PRISMA diagram of literature search and inclusion process. PRISMA, preferred reporting items for systematic reviews and meta-analysis

The PRISMA diagram of literature search and inclusion process is shown in Fig. 1 [16]. In total, 21 studies were included in the systematic review in which 11 different RATs for prolonged QT were used [12, 14, 19–24, 31–33, 36, 40, 43, 44]. Most commonly used tools were risk scores (*n* = 9) [12, 14, 21–27], computerized physician order entry systems (*n* = 3) [31–33], and clinical decision support systems (*n* = 6) [19, 20, 36–39]. Of the studies, eight were conducted in the USA [12, 21, 22, 32, 33, 36, 38, 44], four in Belgium [14, 23, 24, 27], two in Australia [25, 40, 43], one in Germany [20], four in the Netherlands [26, 31, 37, 39], and one in Sweden [19]. The RATs were developed mainly for physicians and pharmacists. Most of the studies were conducted in inpatient settings. Of the studies, one applied a prospective controlled interventional study design [20], while the majority were observational studies (Supplementary Table S2).

### Comparison of studies including risk scores (n = 9)

Nine studies described a risk score for QT prolongation as a RAT (Table 1; Supplementary Table S2) [12, 14, 21–27]. In all nine studies, ECG data were available [12, 14, 21–27]. The definition of long QT interval differed slightly between the studies (Table 1). The QT interval was corrected for heart rate with three different correction formulae, Bazett’s [28], Fridericia’s [29], and Rautaharju’s correction formulae [30] (Table 1).

**Table 1:** Studies in which risk scores were applied as a risk assessment tool (*n* = 9)

**Study** **Study design** **Sample** **(*n*, age [years, mean, SD])**	**Definition** **of QT prolongation,** **QTc formula used in the study**	**Included risk factors in the risk score**	**Points allocated each risk factor** **Total points of the risk score**	**Points indicating risk category**	**Predictive performance of the risk score**
**Prospective studies**					
**Tisdale et al. **[12] Prospective observational study, Indiana University Health Methodist Hospital, USA Risk score derivation group: 900 patients Age: 65 ± 15 Risk score validation group: 300 patients Age: 65 ± 14 23 patients belonging to both groups	QTc ≥ 500 ms or an increase in QTc of ≥ 60 ms compared with the admitting value at any time during hospitalization Bazett’s correction formula [24]	**Demographic factors:** age > 68 years, female sex **QT affecting clinical conditions and morbidities:** sepsis, heart failure, acute myocardial infarction **Electrolytes:** Potassium levels **ECG parameters**: Admission QTc ≥ 450 ms **Loop diuretics** **QT prolonging drugs ** [1]	The risk score allocated weighted points based on log ORs for each risk factor (1–3 points) Maximum points: 21	**Low-risk category:** < 7 points **Moderate risk category:** 7–10 points **High-risk category:** ≥ 11 points	**Moderate risk category:** Sensitivity: 0.67 Specificity: 0.88 PPV: 0.55 NPV: 0.88 **High-risk category:** Sensitivity: 0.74 Specificity: 0.77 PPV: 0.79 NPV: 0.76
**Vandael et al.** [14] Prospective, observational study, University Hospitals Leuven, Belgium 178 patients Age: 69 ± 14 (range 20–96)	**Moderately prolonged:** QTc ≥ 450–500 ms (men) or QTc ≥ 470–500 ms (women). **Severely prolonged:** QTc ≥ 500 ms Fridericia [25] and Rautaharju [26] correction formulae	**Preliminary RISQ-PATH score:** **Demographic factors:** Age ≥ 65 years, female sex, smoking, body mass index **QT affecting clinical conditions and morbidities:** (ischemic) cardiomyopathy, hypertension, arrhythmia, thyroid disturbances, liver failure, neurological disorders, diabetes mellitus **Electrolyte disturbances:** potassium, calcium levels **Lab results:** CRP > 5 mg/l, GFR ≤ 30 ml/min **ECG parameters**: prolonged QTc on baseline ECG **QT prolonging drugs:** allocated points according to the QTDrugs Lists of CredibleMeds [1]	Points allocated according to the evidence level of the risk factors (evidence from a systematic review [4]); 0.5, 1, 3, or 6 points Drugs of CredibleMeds [1] QTDrugs List 1 (known risk of TdP) allocated 3 points Drugs of QTDrugs List 2 (possible risk of TdP) allocated 0.5 points Drugs of List 3 (conditional risk of TdP) allocated 0.25 points Maximum points: 40.5 points + sum of QT drugs	The cutoff value of 10 points was set as high risk for QT prolongation	RISQ-PATH score < 10: **Sensitivity:** 96.2% (95% CI 78.4–99.8%) **Specificity:** 32.9% (95% CI 25.6–41.0%) **PPV:** 19.7% (95% CI 13.4–27.9%) **NPV:** 98.0% (95% CI 88.2–99.9%) Without baseline ECGs included, the **NPV** and **sensitivity** were 94.3% (95% CI 83.4–98.5%) and 88.5% (95% CI 68.7–97.0%), respectively
**Retrospective studies**					
**Haugaa et al. **[21] Retrospective study, Mayo Clinic, USA 470 patients with isolated QTc ≥ 500 ms Age: 55 ± 24	QTc ≥ 500 ms Bazett’s correction formula	**Pro-QTc score:** **Demographic factors:** Female sex **QT affecting clinical conditions and morbidities:** acute coronary syndrome, anorexia nervosa or starvation, bradycardia, cardiac heart failure, diabetes mellitus (I, II), hypertrophic cardiomyopathy, hypoglycemia, intoxication of QT prolonging drugs, long QT syndrome, pheochromocytoma, renal dialysis, stroke, and head trauma. Status after atrial fibrillation, cardiac arrest, and syncope or seizure **Electrolyte disturbances:** hypocalcemia hypokalemia, hypomagnesemia **QT prolonging drugs** from the QTDrugs Lists of CredibleMeds [1]	Pro-QTc score created from the sum of QT prolonging factors. Each risk factor was considered equal and each risk factor was allocated 1 point, owing to lack of specific data for each point Total points that could be allocated were not specifically mentioned	A risk score of ≥ 4 indicated higher mortality HR: 1.72 (95% CI, 1.11–2.66; *p* < 0.001)	-
**Joyce et al. **[22] Retrospective study, Mayo Clinic, USA Post-op: 59 patients Age: 62 ± 21 Patients with alerted ECG (*n* = 411) Age: 54 ± 25	QTc ≥ 500 ms	See Haugaa et al. [21]	See Haugaa et al. [21]	A risk score of ≥ 4 indicated higher mortality	-
**Vandael et al. **[23] Retrospective study, University Hospitals Leuven, Belgium 222 patients Age: 77.3 years (range 23.7–97.2)	**Moderately prolonged:** QTc ≥ 450–500 ms (men) or QTc ≥ 470–500 ms (women). **Severely prolonged:** QTc ≥ 500 ms Bazett’s correction formula ^28^	**Demographic factors:** female sex, body mass index **QT affecting clinical condition and morbidities:** bradycardia, myocardial infarction, heart failure, syncope, hypertrophic cardiomyopathy, atrial and ventricular rhythm disturbance, other rhythm disturbances, diabetes mellitus, CNS disorders, renal dialysis **Electrolyte disturbances:** hypokalemia **Additional QT prolonging drugs** besides haloperidol	Each risk factor counts for 1 point Maximum points: 14	A risk score of ≥ 4 indicated higher mortality	-
**Vandael et al.** [24] Retrospective study, University Hospitals Leuven, Belgium 19 TdP cases Age: 74 ± 12 (range 47–87)	See Vandael et al. [14]	Preliminary RISQ-PATH score — see Vandael et al. [14]	See Vandael et al. [14]	See Vandael et al. [14]	-
**Buss et al. **[25] Retrospective study, 500 medication review reports from Australian pharmacists The risk of drug-induced QT interval prolongation was calculated for 325 patients Age: 76 ± 12 (range 20–97)	See Vandael et al. [14]	Preliminary RISQ-PATH score—see Vandael et al. [14]	See Vandael et al. [14]	See Vandael et al. [14]	-
**Bindraban et al. **[26] Retrospective, descriptive study, Spaarne Gasthuis Hospital, The Netherlands The objective was to develop and validate a risk model to predict QTc interval prolongation of eligible ECGs for patients using QTc prolonging drugs 19,340 ECGs, recorded in 6927 patients Age: 71.7 Development set: 12,949 ECGs (5685 patients) Validation set: 6391 ECGs (3721 patients)	QTc ≥ 500 ms Bazett’s correction formula ^28^ was used	**Complete model included:** **Demographics:** gender, age > 70 years **QTc prolonging drugs**: antidiabetic drugs, antiarrhythmics, acetylsalicylic acid, loop diuretics, thyroid hormones, beta-blockers, verapamil/diltiazem, the number of prescriptions of QTc prolonging drugs **Electrolyte disturbances**: potassium, calcium, magnesium **Lab results**: ALAT, eGFR **The maximum QTc time measured in the last 365 days before ECG recording** **Simplified model: the variables calcium level, magnesium level, and maximum QTc interval measured in the past 365 days were excluded**	The risk factors with the accompanying risk score (1–7 points) were included in the risk model with binary logistic regression Maximum points: 24 in complete model and 18 in simplified model (excluding calcium, magnesium, and the maximum QTc time measured in the last 365 days)	The performance was best and the specificity and sensitivity highest with a cutoff value of ≥ 5 Points indicating different risk categories were not demonstrated and cutoff value should be set before implementing the risk model in medication surveillance system	(Overall Sensitivity: 0.81 Specificity: 0.48) With a cutoff value of ≥ 5 Complete model: Sensitivity: 0.63 Specificity: 0.69 PPV: 0.14 NPV:0.96 Accuracy: 0.68 Simplified model: Sensitivity: 0.48 Specificity: 0.73 PPV: 0.12 NPV:0.95 Accuracy: 0.71
**Vandael et al. **[27] Retrospective study, the Nexus Hospital Network (*n* = 17), in Belgium 60,208 patients Age: 63 ± 18	**Moderately prolonged:** QTc ≥ 450–500 ms (men) or QTc ≥ 470–500 ms (women). **Severely prolonged:** QTc ≥ 500 ms Fridericia (for QRS < 120 ms) or Rautaharju correction formulae (for QRS ≥ 120 ms) (Vandenberg et al. 2016 [47])	See Vandael et al. [14] The aim was to optimize the RISQ-PATH score [14], by improving the weights allocated to each factor and subsequently to validate this score in a large database of patients. The main focus was to acquire a high sensitivity (> 85%) while maximizing the specificity of the risk score Multiple logistic regression was conducted in development of the RISQ-PATH model. Risk factors excluded from the original RISQ-PATH score: diabetes, number of drugs in list 2 of CredibleMeds, thyroid disturbances	-	-	The optimized RISQ-PATH model has an area under the ROC curve of 0.772 (95%CI 0.763–0.780) to predict QTc ≥ 450(♂)/470(♀) ms. A predicted probability of ≥ 0.035 was set as cutoff for a high risk of QTc prolongation Sensitivity: 0.874 (95% CI 0.862–0.885) Specificity: 0.462 (95% CI 0.458–0.466) PPV: 0.092 NPV:0.983

*SD* standard deviation, *OR* odds ratio, *PPV* positive predictive value, *NPV* negative predictive value, *HR* hazard ratio, *CI* confidence interval

In addition to QT prolonging drugs, the risk scores included non-pharmacological risk factors for QT prolongation such as demographic factors, QT interval affecting clinical conditions, and morbidities and electrolyte disturbances (Table 1). QT prolonging drugs were mainly identified using the QTDrugs Lists of CredibleMeds [1, 12, 14, 21–27]. Tisdale et al. [12] stated that evidence of QT prolonging drugs was taken from published trials and case reports, and CredibleMeds [1] was a used source. Included QT interval affecting diagnoses differed between the risk scores used in the studies (Table 1). The original RISQ-PATH score by Vandael et al. [14, 24] contained the highest number of risk factors, obtained from a systematic review [4], and the least number of co-morbidities was found in the risk score by Tisdale et al. [12]. The RISQ-PATH score also included risk factors that have not been validated as risk factors (hypertension, cigarette smoking, obesity, and others).

A limitation of five of the studies [21–25] was the lack of information on predictive performance (Table 1). The predictive performance was presented in four studies (Table 1) [12, 14, 26, 27]. In the study on the pro-QTc score, an institution-wide QT alert system for assessment of mortality was developed [21]. The system screened all ECGs performed and alerted physicians if finding QTc ≥ 500 ms. The risk scores by Vandael et al. [14, 23, 24, 27] were developed in various hospital wards with different specialties, which makes them more generalizable than the risk score by Tisdale et al. [12], developed in cardiac critical care units. Selection bias may be present in some of the studies [12, 21–23]. The RISQ-PATH score developed by Vandael et al. [14] consists of many predictors that are frequently not available, which may challenge the use in clinical practice. The risk of QT prolongation or TdP may have been underestimated in the studies including the preliminary RISQ-PATH score due to missing lab results, exclusion of patients, or unrecognized TdP cases because of lack of information in the patient files or wrongly coded cases [14, 24]. In a later study by Vandael et al. [27], the RISQ-PATH score was optimized and validated in a large patient cohort, and an algorithm was proposed to be used in clinical decision support systems to create smart QT alerts. Risk factors excluded from the original RISQ-PATH score [14] were diabetes, number of drugs in list 2 of CredibleMeds, and thyroid disturbances.

Bindraban et al. [26] developed and validated two risk models (complete and simplified), both having a lower number of risk factors than the RISQ-PATH score [14]. Risk models used variables that usually are automatically available in healthcare information systems, and therefore, the risk models could be implementable in a clinical decision support system.

### Comparison of studies on computerized physician order entry systems (n = 3)

Computerized physician order entry systems (CPOE), used to reduce the risk of QT prolongation and related morbidities, were studied by van der Sijs et al. [31], Muzyk et al. [32], and Sorita et al. [33] (Table 2; Supplementary Table S2). The studies did not measure outcomes such as occurrence of cardiovascular illness, TdP, or mortality. The retrospective studies [31, 32] used small sample sizes; thus, their generalizability is limited and selection bias probable. The quasi-experimental study [33] was conducted in an institution-wide setting and could not exclude confounding factors and dependency of data due to the study design [33]. The CPOE QT alert override rate was 77%.

**Table 2:** Studies using computerized physician order entry systems (CPOE) as a risk assessment tool (*n* = 3)

Study Study design Setting Sample (*n*, age [years, mean, SD])	Study description	Source of evidence on QT prolonging drugs	Results of the study
**van der Sijs et al.** [31] Retrospective study 49 patients, 59% of patients > 65 years Erasmus University Medical Center, the Netherlands	Investigation of a CPOE including the Dutch national drug database with DDI alerting on QT prolongation The study investigated whether adjustment to a later version would improve the identification of patients at risk of developing TdP (version from 2005 vs 2007) The system was updated after complaints of too many low-specificity DDI alerts on QT prolongation	Version 2005: lists by De Ponti [34, 35] and all class Ia and III antiarrhythmics generated alerts Version 2007: evidence of QT prolonging drugs was taken from CredibleMeds [1]	Fifteen (31%) patients were at risk of TdP, and these patients used two QT prolonging drugs. The updated system introduced a sensitivity problem; for 53% of the patients considered at risk of TdP, no QT alert was generated The updated system generated 23 alerts instead of 49 alerts. With a sensitivity of 47%, assuming the old system identified all patients at risk of TdP development. However, the PPV remained low; the PPV in the old version was 31% and in the updated version 30%. The accuracy of the QT alert generation in the CPOE was low, since it depended on drug classes and not patient related factors
**Muzyk et al. **[32] Retrospective study **Pre-CPOE set group:** 84 patients receiving intravenous haloperidol Age: 62.5 ± 19.3 **Post-CPOE set group:** 67 patients receiving haloperidol Age: 64.8 ± 18.7 Duke University Hospital, USA	Investigation of the effects of implementing a CPOE set on adherence to monitor parameters, maximum and cumulative doses, and identification or mitigation of risk factors for QT prolongation in patients prescribed intravenous haloperidol	QT prolonging drugs were identified based on the QTDrugs Lists of CredibleMeds [1]	Fewer patients received a 24-h cumulative haloperidol dose of ≥ 2 mg in the post-CPOE set group than in the pre-CPOE set group (47.8% vs 64.3%, *p* < 0.048) Patients in the post-CPOE set group were monitored with ECG more often and were more likely to have an ECG following administered intravenous haloperidol (61.2% for the post-CPOE set group vs 39.3%, *p* = 0.009). In the post-CPOE set group, ECG monitoring 24 h after a haloperidol dose was conducted more often than in the pre-CPOE set group (58.5% vs 25.2% of the time) Rates of concomitant QTc prolonging drugs were similar between groups at approximately 50%. The CPOE included a link to information on QT prolonging drugs. After implementation, the link did not help in decreasing concomitant use of QT prolonging drugs
**Sorita et al.** [33] Quasi-experimental study **Silent phase:** 359 patients Age: 64.2 ± 18.7 **Active phase:** 648 patients Age: 63.7 ± 19.1 110 patients belonging to both groups Mayo Clinic, USA	Evaluation of efficacy after development and implementation (active phase) of a “CPOE QT alert” (clinical decision support) that was triggered when a torsadogenic drug was attempted to be prescribed to patients with documented QT prolongation, found through the QT alert system by Haugaa et al. [21]	QT prolonging drugs identified based on the lists “known risk of TdP” and “possible risk of TdP” of CredibleMeds [1]	The proportion of completed orders for QT prolonging drugs was reduced after the CPOE QT alert system was activated (16.8% [95% CI 14.7–18.9%, *p* < 0.001]) Across all specialties, all provider types, and education levels in the clinic, a significant reduction in orders was seen after the activation of the system. Ordering attempts were less likely to be completed after the activation, OR 0.18 (95% CI 0.14–0.23, *p* < 0.001)

*SD* standard deviation, *DDI* drug-drug interaction, *PPV* positive predictive value, *CI* confidence interval, *OR* odds ratio

### Comparison of studies on clinical decision support systems (n = 6)

Clinical decision support systems (CDSS) were studied in six studies [19, 20, 36–39] (Table 3; Supplementary Table S2). The interventional study by Bertsche et al. [20] investigated drug-drug interactions (DDIs) and DDI-related adverse drug events in intensive care patients with a CDSS (Table 3). The content of the CDSS was developed by an interdisciplinary team with systematic literature searches. Randomization and blinding were not stated in the article; therefore, selection bias cannot be excluded.

**Table 3:** Studies researching clinical decision support systems (CDSS) (*n* = 6)

Study Study design Setting	Sample (*n*, age [years, mean, SD])	Study description	Results
**Bertsche et al. **[20] Prospective controlled cohort study, consecutive design Medical intensive and intermediate care unit in a university hospital, Germany	Patients with ≥ 8 drugs concurrently prescribed, based on pilot study **Control group:** 136 patients Age: 61.0 ± 15.2 **Intervention group:** 129 patients Age: 61.9 ± 14.9 Of these, 57 patients remained in the control group and 53 patients remained in the intervention group until day 7 after admission	Investigation of DDIs and DDI-related ADEs in 265 patients with a developed and pilot-tested CDSS containing information on risk and management of 9453 drug combinations In the control phase, only life-threatening DDIs and contraindications from the CDSS were forwarded to a senior clinician In the intervention phase, information from the CDSS was approved by a pharmacist and forwarded to a senior clinician, 3 days after patient admission. ADRs were observed until day 7 after admission, transfer to other units, discharge, or death. DDI warnings were only given on day 3	DDIs appeared more frequently in controls than in the intervention group (66 vs 54%, *p* = 0.02, RRR: 18%). The percentage of patients with at least 1 DDI-related ADR was lower in the intervention group (25%) than in the control group (44%) until day 7 after admission (*p* < 0.01, RRR: 43%), mainly due to QTc prolongation and hypokalemia incidence reduction The incidence of QT prolongation was reduced by 64% from 15 (11%) patients in the control group to 5 (4%) in the intervention group (*p* = 0.04) QTc prolongation was predicted as a possible DDI for 31 drug pairs in the control group, QTc prolongation occurring in 19 (61%) of them QT prolongation was predicted in 42 drug pairs in the intervention group and occurred in 10 (24%) of them (*p* < 0.01, RRR: 61%) Physicians discontinued a drug twice as often after a DDI alert due to the intervention. In the intervention, fewer patients needed a prescription for new medication to treat ADRs (OR: 0.55, *p* < 0.02)
**Tisdale et al. **[36] Prospective observational study Cardiac care units (CCU), Indiana University Health Methodist Hospital, USA	**Pre-intervention group:** 1200 patients Age: 48% > 67 years **CDSS implementation group:** 1200 patients Age: 39% > 67 years	Investigation of the effectiveness of a CDSS with an incorporated risk score [12] for reducing the risk of QT prolongation. The CDSS alerted pharmacists entering orders for QT prolonging drugs, who could then discuss risk mitigation strategies with the prescriber (1) Pre-intervention: data collection in pre-intervention group, development, and validation of a risk score [12] (2) Development and modification of the CDSS. Incorporation of the risk score [12]. The CDSS was shown if a QT prolonging drug was ordered; the patient had a moderate- or high-risk score [12] or admitting QTc > 500 ms. Pharmacy and physician staff were educated about the system (3) Intervention testing: data collection in CDSS implementation group, assessment of the CDSS, impact of the CDSS	CDSS implementation resulted in a reduced risk of QT prolongation (adjusted OR: 0.65, 95% CI 0.56–0.89, *p* < 0.001). A reduction in the prescribing of torsadogenic non-cardiac medications was seen after implementation of the CDSS (adjusted OR: 0.79; 95% CI 0.63–0.91, *p* = 0.03) The percentage of patients with a high-risk score was lower after the implementation of the CDSS (4.4% vs 10.3%, *p* < 0.001), while the percentage of patients with a moderate risk score was higher (41.1% versus 35.5%, *p* = 0.003) The proportion of patients with QT prolongation associated with medications was lower after implementation of the CDSS than in the pre-intervention phase (9.7% vs 16.9%, *p* < 0.001)
**Böttiger et al. **[19] Prospective 4-month pilot study and surveys before and after Two geriatric wards, three primary healthcare centers, Sweden	**Pilot study:** 503 patients from geriatric wards and 368 from primary care **Surveys:** Pre-study questionnaire respondents: 32 primary care physicians and 29 geriatricians 2nd questionnaire after 4 months from starting to use the CDSS: Results are based on responses of 17 primary care physicians and 15 geriatricians who had actually used the CDSS	Development of PHARAO, a CDSS presenting a risk profile for adverse events of drugs. 1427 substances scored in relation to their risk to cause any of nine adverse events, including QT prolongation/arrhythmia For QT prolongation, the substances were scored from 0 (no pharmacological effect) to 3 (strong pharmacological effect). Algorithms for each adverse event score were developed to create individual risk profiles	The study found that 136/1427 substances were classified for arrhythmic properties. In patients in geriatric wards (*n* = 503), high-risk signals regarding QT prolongation/arrhythmia appeared in ~ 10% and in primary care patients (*n* = 368) in ~ 5% PHARAO was considered easy to use and supported medication review by most physicians. The physicians learned about side effects of drugs. 21/32 physicians would recommend PHARAO, another 5 if PHARAO was modified
**Berger et al. **[37] Prospective, observational study	**Development cohort**: 107 patients Age: 56.0 (median) **Validation cohort**: 1579 patients Age: 77.0 (median)	A model was developed based on risk factors associated with QTc prolongation determined in a prospective study on QT-DDIs in a university medical centre in the Netherlands. The main outcome measure was QTc prolongation defined as a QTc interval > 450 ms for males and > 470 ms for females. Review from literature was conducted on additional risk factors The ability of the model to predict QTc prolongation was validated in an independent dataset obtained from a general teaching hospital against QTc prolongation as measured by an ECG as the gold standard	The model included the following risk factors (each having scores 1 or 2): age, gender, cardiac comorbidities, hypertension, diabetes mellitus, renal function, potassium levels, loop diuretics, and QTc-prolonging drugs (according to CredibleMeds [1]) Application of the model resulted in an area under the ROC curve of 0.54 (95% CI 0.51–0.56) when QTc prolongation was defined as > 450/470 ms, and 0.59 (0.54–0.63) when QTc prolongation was defined as > 500 ms. A cutoff value of 6 led to a sensitivity of 76.6 and 83.9% and a specificity of 28.5 and 27.5%, respectively
**Chernoby et al. **[38] A multicenter, retrospective quasi-experimental study Ascension Southeast Michigan, consisting of 5 community teaching hospitals that use a common EMR and drug interaction platform	Patients with a known risk of TdP with a documented QTc greater than 500 ms **Silent phase** (for testing purpose of the QT-CDS): 49 patients Age: 67.3 (15.1) **Active phase:** 100 patients Age: 66.2 (15.8)	A QT-CDS tool was implemented, and the study was conducted to evaluate provider response to CDS alerts. The primary outcome was the proportion of orders triggering QTc alerts that were continued without intervention in the active phase compared to the silent phase During the silent phase, clinicians used the existing process with weaknesses: DDI alerts were generated only when 2 or more QTc-prolonging drugs were prescribed (even if a patient´s QTc was greater than 500 ms), and access to the ECG report in the EMR required the activation of 3 to 5 additional screens The QT-CDS was designed to fire an alert each time a prescriber attempt to order a QTc prolonging drug in a patient with QTc greater than 500 ms. A copy of the most recent ECG report could be displayed right from the alert The risk of developing QTc prolongation was calculated using a previously validated scoring system of Tisdale et al. [12]	Implementation of the QT-CDS led to a dramatic reduction in the proportion of QTc alert–generating medication orders continued with no intervention (from 81.6% in the silent phase to 37% in the active phase, an absolute reduction of 54.6%) The traditional drug interaction alert did not result in any orders being discontinued in the silent phase; however, 48% of alert-generating orders were discontinued in the active phase after display of the QT-CDS. The medications most commonly discontinued in the active phase were ondansetron (38.7% of orders), ciprofloxacin (20.8%), and azithromycin (10.4%). Continuation of orders along with an intervention (e.g., electrolyte replacement) occurred at similar rates in the 2 study phases (18% in the silent phase and 15% in the active phase)
**Berger et al. **[39] Intervention study using a pre- and post-design in 20 community pharmacies in the Netherlands	All QT-DDIs including QTc-prolonging drugs with a known risk of TdP that occurred in the community pharmacies (*n* = 20) during a study period of 3 months before and 3 months after CDS tool implementation	The use of the CDS tool (consisting a paper-based flowchart) was implemented to study the impact on the handling of QT-DDIs The QTc-prolonging drugs involved in the QT-DDIs are listed at the CredibleMeds [1] For all QT-DDIs, the following variables were collected: the management of the QT-DDI including interventions, the interacting drugs, and the dosages of them For all patients: age, gender, and comorbidities were collected. The following lab values were collected, if registered in Pharmacom®: renal function, liver function, and electrolyte serum levels	A total of 928 QT-DDI alerts were generated during the pre- and post-CDS tool phases **In before period:** unique patients *n* = 233 (median age 66) and QT-DDI alerts *n* = 244, **In after period**: unique patients *n* = 149 (median age 63) and QT-DDIs *n* = 157 There was no significant difference in the proportion of QT-DDIs for which an intervention was made after implementing the tool: 43.0% before and 35.7% after implementation (OR 0.74; 95% confidence interval 0.49–1.11). Substitution of interacting agents was the most frequent intervention. Pharmacists spent 20.8 ± 3.5 min (mean ± SD) on handling QT-DDIs pre-CDS tool, which was reduced to 14.9 ± 2.4 min (mean ± SD) post-CDS tool. Of these, 4.5 ± 0.7 min (mean ± SD) was spent on the CDS tool

*SD* standard deviation, *DDIs* drug-drug interactions, *ADEs* adverse drug events, *ADRs* adverse drug reactions, *RRR* relative risk reduction, *OR* odds ratio, *CI* confidence interval

Tisdale et al. [36] investigated the effectiveness of a CDSS including a risk score [12], for reducing the risk of QT prolongation (Table 3). The pre-intervention group and the implementation group differed significantly in some respects producing a risk of selection bias. Both the CDSS [36] and the included validated risk score [12] were developed in cardiac care units, limiting the external validity. Another limitation was the alert fatigue, as the override rate was 82%. Of all alerts triggered, 13% resulted in additional patient monitoring and 18% resulted in discontinued mediation orders.

Böttiger et al. [19] described the development of PHARAO (Pharmacological Risk Assessment Online), a CDSS presenting a risk profile for adverse events associated with combined effects of concomitantly used drugs (Table 3). A multidisciplinary team searched pharmacological handbooks, summaries of product characteristics, evaluations from European Medicines Agency, and articles through PubMed for evaluation and scoring of 1427 substances. PHARAO did not use pop-ups to alert physicians. To decrease alert fatigue, a restrictive approach was taken to what drug pairs would be included.

Berger et al. [37] developed a tool based on risk factors associated with QTc prolongation determined in a prospective study on QT-DDIs in a university medical center in the Netherlands. The main outcome measure was QTc prolongation defined as a QTc interval > 450 ms for males and > 470 ms for females. Review from literature was conducted on additional risk factors.

Chernoby et al. [38] conducted a retrospective study to evaluate provider response to CDS alert. The tool used the risk score by Tisdale et al. [12]. The primary outcome was the proportion of orders triggering QTc alerts that were continued without intervention in the active phase compared to the silent phase. The proportion reduced 55%.

Berger et al. [39] studied the use of the CDS tool (consisting a paper-based flowchart) to assess the risk of QT drug-drug interactions in community pharmacies in the Netherlands. All QT-DDIs that occurred during a pre- and post-CDS tool period of 3 months were included. The QTc-prolonging drugs involved in the QT-DDIs are listed at the CredibleMeds. Pharmacist intervened in 43.0% and 37.5% of the QT-DDIs pre- and post-CDS tool.

### Risk assessment tools focusing on ECG parameters (n = 3)

The QT nomogram [40], the ½ RR rule [43], and the T-wave analysis software [44] were developed in retrospective studies. The RATs [40, 43, 44] focused on ECG parameters.

Chan et al. [40] developed a QT nomogram, based on a cloud diagram [41], for assessing the risk of TdP in a retrospective case-controlled study. Cases of drug-induced TdP (*n* = 130) were found in a systematic review (329 full-text articles reviewed) [40]. Controls (*n* = 318) were patients overdosing on non-cardiotoxic drugs, obtained from a previous study [42]. The systematic review included in the retrospective case-controlled study lacked description of included studies and assessment of risk of bias [40].

QT interval and heart rate combinations of cases and controls were plotted with the QT nomogram [40]. For comparison, two curves were plotted corresponding to Bazett’s correction formula [28] at QTc values of 440 ms (medium-risk value) and 500 ms (high-risk value). The validity of some case points of tachycardia was questionable. The sensitivity and the specificity of the QT nomogram and Bazett’s QTc [28] can be seen compared to the ½ RR rule [43] and Fridericia’s QTc [29] in Table 4.

**Table 4:** Sensitivity and specificity of the QT nomogram [40] and the ½ RR rule [43] compared to QT correction formulae

Method	Sensitivity, % (95% CI)	Specificity, % (95% CI)
QT nomogram [33]	96.9 (93.9–99.9)	98.7 (96.8–100)
QT nomogram (cases with heart rate > 104 bpm excluded) [33]	98.3 (96.1 − 100)	99.3 (97.8 − 100)
½ RR rule [36]	87.6 (80.4–92.5)	52.9 (47.2–58.4)
½ RR rule ≥ 60 bpm [36]	100 (94.6–100)	49.7 (43.8–55.5)
Bazett’s QTc = 440 ms [33]	98.5 (96.4–100)	66.7 (58.6–74.7)
Bazett’s QTc = 500 ms [33]	93.8 (89.6–98.0)	97.2 (94.3–100)
Fridericia’s QTc > 500 ms [36]	82.2 (75.6–88.8)	100 (100–100)

The ½ RR rule defines an abnormal QT interval as a QT greater than half of the RR interval on an ECG and does not require heart rate correction [43]. Berling and Isbister [43] conducted a study for determination of the sensitivity and specificity and comparison of the ½ RR rule with other methods (Table 4). The obtainment of TdP cases (*n* = 129) and controls (*n* = 316) for their study from the literature was described by Chan et al. [40]. Additionally, the study by Berling and Isbister [43] calculated the agreement in eight different sample sets of QT-heart rate pairs from psychotropic medication overdoses. The ½ RR rule [43] and the compared QT nomogram [40] both had poor negative and positive agreement, of which the latter was worse [43]. The ½ RR rule misclassified patients without QT prolongation. Data was collected prospectively [43]. The sensitivity and specificity may be biased for all methods in Table 4 since the cases of TdP were taken from the literature.

Sugrue et al. developed a computer-based repolarization measurement tool to identify electrocardiographic predictors of torsadogenic risk [44]. The software provided extraction of information from automatic 12-lead ECGs. A retrospective T wave analysis of TdP cases and controls using prescribed dofetilide and sotalol was conducted in Mayo Clinic, USA. The sample size was small (*n*_cases_ = 13, *n*_controls_ = 26). There is potential for confounding by certain clinical factors that differed between the groups, as only sex and age were matched (within 2 years). QTc interval discriminated TdP cases from controls in 79%. The sensitivity and specificity for QTc (Bazett’s) alone were 88.1% and 72.0%, respectively. The positive and negative predictive values were 85.0% and 76.9%, respectively. The sensitivity and specificity in predicting TdP was 79.7% and 46.0%, respectively, for the T wave right slope. The positive and negative predictive values were 58.9% and 70.0%, respectively. Adding the T wave right slope in the analysis with QTc, the discrimination increased to 88%.

## Discussion

To our knowledge, this is the first systematic review that summarizes and compares tools for assessing safety risks of patients using QT prolonging drugs. Nine RATs were found of which those applying risk scores were most common, although QT risk assessment was also integrated in some CDSS and CPOE systems. Most of the risk assessment methods were used in inpatient care primarily by physicians and pharmacists. Many of the methods used the QTDrugs Lists of CredibleMeds [1] for identification of QT prolonging drugs. In addition to drugs associated with QT prolongation and TdP, the risk scores considered a wide range of non-pharmacological risk factors [12, 14, 21–27].

The risk scores [12, 14, 21–27] had similarities regarding the design of the risk score and included risk factors. A strength of the RISQ-PATH score [14, 24] was that it included a high number of risk factors and evidence of the included risk factors was assessed in a previously conducted systematic review [4]. Points were allocated according to evidence level [14]. The risk score by Tisdale et al. [12] allocated weighted points based on log odds ratios for each risk factor. As there is lower evidence of certain risk factors and certain drugs associated with QT prolongation and TdP, it is sensible to allocate fewer points to these variables or exclude them completely from the RAT for better predictive performance. The risk score by Tisdale et al. [12] had good predictive performance and was also included in a CDSS [36]. The RISQ-PATH score had high sensitivity and negative predictive value [14]. The risk scores still need to be validated in prospective studies applying various patient groups and larger sample sizes.

The inclusion of patient-specific risk factors for QT prolongation and TdP in CPOE systems and CDSS varied [19, 20, 31–33, 36]. CPOE systems and CDSS could be further developed to minimize alert fatigue [45]. In two included studies, alerts related to the risk for QT prolongation were overridden to a high degree [33, 36]. Two of the studies indicated a reduction in the orders for QT prolonging drugs because of the alerts, but patient outcomes such as TdP were not measured [33, 38]. Böttiger et al. [19] restricted the drug pairs that were included in the CDSS as well as avoided pop-up alerts to avoid a high override rate.

These findings indicate the importance of designing CPOE systems and CDSS with increased sensitivity and specificity and to evaluate them regarding unnecessary generated signals. However, restricting drug pairs seems not to be the only solution to better performance of the RATs. In the updated version of the CPOE in the study by van der Sijs et al. [31], several drugs generating QT alerts were deleted, but the sensitivity problem of the CPOE remained. On the other hand, the RATs including other patient-specific risk factors have been found to excel in outcomes [12, 14, 33, 36]. Thus, further research is needed to optimize the tools for identifying clinically significant risks and patients at risk for QT prolongation and TdP. Future research should also be extended to cover the evaluation of impact of these tools on patient outcomes.

QT correction formulae were not assessed as RATs in our systematic review, but they were mentioned for studies using a correction formula. QT correction formulae have been previously studied [46, 47]. Many of the studies included in our systematic review used Bazett’s correction formula [12, 19, 21, 23, 26, 28, 32, 36, 38, 40, 43, 44]. Bazett’s correction formula has a problem with undercorrection and overcorrection of the QT interval [45]. Fridericia’s correction formula seems to have better performance [47]. This formula was used in five of the included studies [14, 24, 27, 37, 43].

The QT nomogram performed better than Bazett’s correction formula [40]. However, the QT nomogram [40], the ½ RR rule [43], and the T wave analysis tool [44] did not include other patient-specific risk factors than ECG parameters. The QTc is an indicator of risk of TdP, but it does not identify all risks [6, 11, 12]. If these RATs are used, patient-specific risk factors need to be assessed along with other RATs or based on clinical judgment. A survey from 2005 showed that the majority of the healthcare practitioners responding to the questionnaire could not correctly identify factors and drugs that may prolong the QT interval [49]. The T wave analysis tool [44] has potential as a RAT, but if used without considering QTc, it had lower predictive performance [44]. Further studies are needed of the QT nomogram [40] and the T wave analysis tool [44] that apply various patient groups and larger sample sizes, as well as other QT prolonging drugs.

Overall, the RATs may increase safety of patients at risk of QT prolongation or TdP as the studies either showed that a wide range of patient-specific risk factors were considered: ordering of QT prolonging drugs decreased, monitoring of patients increased, advice was given, or healthcare providers were trained about risks associated with QT prolongation. As older adults have an increased risk for QT prolongation and TdP due to the presence of multiple risk factors [6, 7], RATs, considering a wide range of patient-specific risk factors, may be useful when contemplating the use of QT prolonging drugs with these patients. Based on this systematic review, various RATs may be used in combination, e.g., risk scores for QT prolongation and TdP may be incorporated into CDSS and CPOE systems for easier access and risk assessment. Using RATs in combination may also identify high-risk patients from low risk patients and may reduce unnecessary alerts.

This systematic review may assist in the decision to select and use RATs for QT prolonging pharmacotherapy. The included RATs could be further developed to fit different health information systems. There is potential for the RATs to be adapted to outpatient settings and furthermore assist other healthcare providers other than physicians, e.g., community pharmacists [19, 39]. However, no guidelines for managing risks of QT prolongation in primary care or community pharmacies exist and more training on the topic is needed to adopt the tools in clinical practice [48, 50].

The review was conducted according to the PRISMA guidelines [15, 16]. In the process, the GRADE approach was considered [18]. In the search process, the included studies found in PubMed were searched for additional studies. Since only duplicates were found in the search of the Scopus database, another database could possibly have been searched. Most of those included were observational studies, which are more prone to bias because of their design [51]. However, since TdP occurs rarely [40, 52], an observational study design can be useful in finding these events. Clinical validation of QT-RATs with RCT studies is needed, as our systematic review shows that studies are mainly observational or quasi-experimental studies and RCT studies were not found. A comprehensive bias assessment using a bias assessment tool would be useful in future studies, as it was not conducted in this study. Risk of bias assessment was conducted only by the authors, considering bias assessment presented in the included studies.

## Conclusions

Most of the RATs for QT prolonging pharmacotherapy give a comprehensive overview on patient-specific risks of QT prolongation and TdP and reduce modifiable risk factors and actual events. There is potential for the RATs to be adapted to different health information systems in inpatient and outpatient settings. Studies on outcomes of using individual RATs and combining various tools and clinical validation of QT-RATs with RCT studies are needed.

## Supplementary Information

Below is the link to the electronic supplementary material.

Supplementary file1 (DOCX 56 KB)

## Data Availability

All data generated or analyzed during this study are presented in this manuscript.
