# Supplementary material for: Risk assessment tools for QT prolonging pharmacotherapy in older adults: a systematic review
Source: Eur J Clin Pharmacol. 2022 Feb 14;78(5):765–79. doi: 10.1007/s00228-022-03285-3 (PMC9005415; doi:10.1007/s00228-022-03285-3)
Supplement: Supplementary file 1 — Supplementary file1 (DOCX 56 KB) [file 228_2022_3285_MOESM1_ESM.docx]

# Supplementary information

Supplementary Table S1. Databases and search terms used in the systematic literature search.

|  | **PubMed** | **Scopus** |
| --- | --- | --- |
| **Search terms** | (torsades de pointes) OR (torsade de pointes) OR (long QT syndrome) OR (pharmacotherapy) OR (drugs) AND (risk assessment) OR (risk management) OR (risk tool) OR (risk score) OR (risk database) AND (QT prolongation) OR (QT interval) OR QTc) AND (elderly) OR (aged) OR (geriatric) | (risk*AND assess*) OR (risk* AND manag*) OR (risk* AND tool*) OR (risk* AND scor*) OR (risk* AND database*) AND (QT AND prolong*) OR (QT AND interval*) OR (QTc) AND (elder*) OR (aged) OR (geriatr*) AND (pharmacotherap*) OR (drug*) OR (medic*) OR (torsade* AND de AND pointes) OR (long AND QT AND syndrome*) AND NOT INDEX (medline) |
| **Filters** | Publication date from 2005/01/01 to 2017/12/31  Update of the search:  2008/01/01 to 2021/08/09 | Publication year: 2005-2017  Update of the search:  2008/01/01 to 2021/08/09 |

Supplementary Table S2. Summary table of studies included in the systematic review according to the type of the tool, presented in a chronological order (n=21).

| **Author,**  **Country of origin** | **Study design, Study setting** | **Sample and findings** | **Definition of prolonged QT interval** | **QT correction formula** | **Description of study/risk assessment tool** | | | **User** |
| --- | --- | --- | --- | --- | --- | --- | --- | --- |
|  |  |  |  |  | **Description** | **Strengths** | **Limitations** |  |
| **Risk scores (n=9)** | | | | | | | | |
| Haugaa et al. (2013)^21^  USA | Retrospective study.  Mayo Clinic in Rochester,  Minnesota. | Pediatric and adult patients analyzed separately.  Of the 52,579 patients monitored with an ECG^a^, 1,145 patients’ ECGs were alerted and of them 470 had an isolated QTc >500 ms. Mean age (SD^b^) of these 470 patients: 55 (±24) years | A QTc ≥500 ms | Bazett’s correction formula^28^ | An institution-wide computer-based QT alert system and a pro-QTc score. The system alerted physicians if QT interval ≥500 ms. All ECGs performed were screened in the system.  The pro-QT score included demographic factors, QT affecting clinical conditions and morbidities, electrolyte disturbances and QT prolonging drugs from the QTDrugs Lists of CredibleMeds^1^ | The pro-QT score was a predictor of mortality, a pro-QT score ≥4 had a HR^c^ of 1.72 (95% CI^d^ 1.11-2.66, *P* < 0.001.  QT prolonging drugs identified from the QTDrugs List of CredibleMeds^1^ | Retrospective study design.  Maximum points of the pro-QTc score not mentioned. Each risk factor in the pro-QTc score was given one point, without considering level of evidence of the risk factors.  Selection bias. | Physicians |
| Tisdale et al. (2013)^12^  USA | Prospective, observational study.  Two Cardiac Critical Care Units, Indiana University Health Methodist Hospital | **Risk score derivation group:** 900 patients.  Age (mean, SD): 65 ± 15 years  **Risk score validation group:** 300 patients.  Age (mean, SD): 65 ± 14 years  23 patients belonging to both groups. | QTc interval ≥500 ms or an increase in QTc of ≥60 ms compared with the admitting value at any time during hospitalization | Bazett’s correction formula^28^ | A risk score was developed in the derivation group and then additionally applied to the validation group.  To each risk factor, based on log ORs^e^, a point was assigned (1-3 points). Risk factors included demographic factors, QT affecting clinical conditions and morbidities, QT prolonging drugs, loop diuretics, potassium levels and QTc interval from ECG.  **Low-risk:** <7 points, **moderate risk:** 7-10 points, **high-risk:** ≥11 points.  Maximum risk score points: 21 | Prospective study including risk derivation group and validation group.  **Predictive performance:**  **Moderate risk category:**  Sensitivity: 0.67  Specificity: 0.88  PPV^f^: 0.55  NPV^g^: 0.88  **High-risk category:**  Sensitivity: 0.74  Specificity: 0.77  PPV: 0.79  NPV: 0.76 | Positive predictive value lower than desired.  Possible selection bias.  Inpatients from 2 Cardiac Critical Care Units, limited the generalizability of the results. | Health care providers in inpatient setting, mainly pharmacists and physicians |
| Joyce et al. (2015)^22^  USA | Retrospective study  Mayo Clinic in Rochester, Minnesota  An analysis of patients whose ECG  provoked an institution-wide QT alert within 24 hours of surgery (post-op) | 470 patients with an isolated QTc ≥500 ms. Of these:  **Post-op:** 59 patients.  Age (mean, SD): 62 ± 21 years.  61 % of post-op cohort receiving QT prolonging medication.  **Patients with alerted ECG** (see Haugaa et al.^21^): 411 patients.  Age (mean, SD): 54 ± 25 years | A QTc ≥500 ms | Not stated | ECGs analyzed by a QT alert system algorithm previously described by Haugaa et al.^21^, and pro-QT score. In this study the score assigned one point for each of these findings: presence of QT affecting clinical diagnosis, QT prolonging electrolyte disturbances, QT prolonging medications present on the QTDrugs Lists of CredibleMeds.^1^ | The research cardiologist was blinded to the QT measurements.  For strengths of the pro-QT score, see Haugaa et al.^21^ | Retrospective study design.  Significant difference in age between the cohorts.  For limitations of the pro-QT score, see Haugaa et al.^21^ | Physicians |
| Vandael et al. (2016)^23^  Belgium | Retrospective study  University Hospitals Leuven | 222 hospitalized patients receiving haloperidol, excluding ICU patients and pediatrics.  Overall median age (range): 77.3 years (23.7-97.2 years) | **Moderately prolonged:**  QTc≥450–500 ms (men) or QTc≥470–500 ms (women). **Severely prolonged:**  QTc≥500 ms | Bazett’s correction formula^28^ | Risk score based on a pro-QTc score by Haugaa et al.^21^  1 point per risk factor (demographic factors, QT affecting conditions and morbidities, hypokalemia). For additional QT prolonging drugs besides haloperidol, one additional point each was gained.  Maximum 14 points.  A risk score of ≥4 linked with a significant higher mortality. | Development of risk scores in various hospital wards. QT prolonging drugs identified from QTDrugs Lists of CredibleMeds^1^ | Retrospective study design.  Every risk factor allocated equal points in the risk score.  Short follow-up period.  Possible selection bias.  Possible overestimation of patients’ risks. | Health care providers in inpatient setting |
| Vandael et al. (2017)^14^  Belgium | Prospective, observational study.  University Hospitals Leuven | 178 hospitalized patients.  Overall mean age (range):  69 ± 14 years (20–96 years) | **Moderately prolonged:**  QTc ≥450–500 ms (men) or QTc ≥470–500 ms (women). **Severely prolonged:**  QTc ≥500 ms | Fridericia^29^ (if QRS <120 ms), Rautaharju^30^ (if QRS ≥120 ms) | RISQ-PATH score.  Points allocated in accordance with the evidence level for risk factors.^4^  Risk factors included demographic factors, QT affecting conditions and morbidities, prolonged QTc on baseline ECG, electrolyte disturbances, lab results. QT prolonging drugs allocated points according to the QTDrugs Lists of CredibleMeds^1^  Maximum 40.5 points + sum QT prolonging drugs.  Cut-off value of 10 points was set as high risk for QTc-prolongation. | High sensitivity  (96.2% [95% CI 78.4–99.8%]) and NPV (98.0% [98.0% (95% CI 88.2–99.9%]) of a risk score <10.  Each risk allocated different points according to evidence level, evidence from a previously conducted systematic review.^4^  Two QT correction formulae were used. | The risk of QT prolongation may have been underestimated due to missing lab result data and exclusion of patients. | Health care providers in inpatient setting |
| Vandael et al. (2017)^24^ | Retrospective study  University Hospitals Leuven, Belgium | 19 TdP cases.  Age (mean, SD): 74 ± 12 years (range 47–87 years) | **Moderately prolonged:**  QTc ≥450–500 ms (men) or QTc ≥470–500 ms (women). **Severely prolonged:**  QTc ≥500 ms | Fridericia^29^ (if QRS <120 ms), Rautaharju^30^ (if QRS ≥120 ms) | See RISQ-PATH score^14^ | See RISQ-PATH score^14^ | Retrospective study design.  Underestimation of TdP due to unrecognized TdP cases because of lack of information in the patient files or wrongly coded cases. | Health care providers in inpatient setting |
| Vandael et al. (2018)^27^  Belgium | Retrospective study  the Nexus hospital network (n=17), Belgium | 60 208 patients  Age (mean, SD): 63± 18 | **Moderately prolonged:**  QTc ≥450–500 ms (men) or QTc ≥470–500 ms (women). **Severely prolonged:**  QTc ≥500 ms | Fridericia^29^ (if QRS <120 ms), Rautaharju^30^ (if QRS ≥120 ms) | The aim was to optimize and validate the previously developed RISQ-PATH score^14^ in a large patient cohort, and to propose algorithm to generate smart QT signals in the electronic medical record. | See RISQ-PATH score^14^. Furthermore, the large number of included patients from different hospital wards.  All risk factors were defined with clear criteria to make it possible to automatically extract these from the EMR.  A strategy (the missing-indicator method) applied to deal with missing values which broadens the applicability of the model. | Integration of pharmacokinetic aspects of drug and information on genetic predisposition might further improve the model  Smoking habits could not be extracted reliably.  Automatic measurement of QT and heart rate were relied.  It was not possible to validate the RISQ-PATH model using clinical events (such as TdP) as hard end-points. | Health care providers in inpatient setting |
| Bindraban et al. 2018^26^  The Netherlands | Retrospective, descriptive study to develop and validate a risk model to predict QTc interval prolongation.  Spaarne Gasthuis hospital, The Netherlands | 19,340 ECGs , recorded in 6927 patients  Age: 71.7  Development set: 12,949 ECGs (5685 patients)  Validation set: 6391 ECGs | **QTc interval > 500 ms** | Bazett´s correction formula^28^ | independent risk factors for QTc interval prolongation  were determined using binary logistic regression. Risk scores were assigned based on the beta coefficient. In the risk-score  validation set, the area under the ROC-curve, sensitivity and specificity were calculated.  Maximum points: 24 in complete model, and 18 in simplified model (excluding calcium, magnesium and the maximum QTc time measured in the last 365 days) | With a cut- off value of ≥ 5  Complete model:  Sensitivity: 0.63  Specificity: 0.69  PPVc: 0.14  NPVd:0.96  Accuracy: 0.68  Simplified model:  Sensitivity: 0.48  Specificity: 0.73  PPVc: 0.12  NPVd:0.95 | ECGs were not reviewed manually.  Missing laboratory values were analyzed as being within the normal range. | Healthcare providers |
| Buss et al. 2018^25^  Australia | Retrospective  study  Australian pharmacies | 500 medication review reports, risk of drug-induced QTc- prolongation was calculated for 325 patients (age: 76 ± 12).  71% of patients used QTc prolonging drugs. Pharmacists provided recommendations only for 23% of patients with a high-risk score and taking drugs with known risk of QT interval prolongation. | **Moderately prolonged:**  QTc ≥450–500 ms (men) or QTc ≥470–500 ms (women). **Severely prolonged:**  QTc ≥500 ms  RISQ-PATH score by vandael et al 2017 was utilized | Not mentioned | The study’s objectives  were to determine the use of QT interval-prolonging drugs in an elderly community-dwelling population at risk of medication misadventure and identify recommendations regarding the risk of QT interval prolongation made by pharmacists when performing medication reviews. |  | Retrospective study design, no opportunities to check the validity of the recorded data | Pharmacists |
| **Computerized physician order entry systems (CPOE) (n=3)** | | | | | | | | |
| van der Sijs et al. (2009)^31^  the Nether-lands | Retrospective study.  Comparison of QT alerts in a CPOE in 2005 and in 2007.  Erasmus University Medical Center,  Rotterdam. | 49 patients.  Age: >59% of patients >65 years | Increased risk of TdP was defined as QTc interval >500 ms or an increase in the QTc interval >60 ms | Not mentioned | CPOE with DDI^h^ alerts.  The hospital CPOE included the national drug database, which introduced DDI alerts on QT prolonging drugs in 2005. In 2007, the QT drugs alert version was updated because of low specificity.  The study investigated whether adjustment to a later version would improve the identification of patients at risk of developing TdP (version from 2005 vs 2007).  For each patient included in the study, interacting drugs, risk factors for TdP and ECG were collected. | The alert version from 2007 used the QTDrugs Lists of CredibleMeds^1^ to classify drugs associated with QT prolongation. | Retrospective study design.  PPV 31% using the QT alert version from 2005 and 30% using the QT alert version from 2007, i.e. the specificity problem remained.  The QT alert version from 2007 introduced a sensitivity problem as it missed 53% of the patients at risk of developing TdP.  Low accuracy of the QT alerting.  Low external validity.  Selection bias. | Physicians in an inpatient setting |
| Muzyk et al. (2012)^32^  USA | Retrospective cohort study  One 32-bed standard care general medical unit in Duke University Hospital, Durham, NC, USA | **Pre-CPOE set group:**  84 patients receiving intravenous haloperidol. Age (mean, SD): 62.5 ± 19.3 years  **Post-CPOE set group:**  67 patients receiving haloperidol.  Age (mean, SD) 64.8 ± 18.7 years | The order set advised cautious use of intravenous  haloperidol in patients with a QTc >500 ms | Bazett’s correction formula^28^ | Investigation of the effects of implementing a CPOE set on adherence to monitor parameters, maximum and cumulative doses, and identification or mitigation of risk factors for QT prolongation in patients prescribed  intravenous haloperidol.  Information on QT prolonging drugs provided through a link in the CPOE. | The QT prolonging drugs were identified based on the QTDrugs Lists of CredibleMeds.^1^  Fewer patients received a 24-hour cumulative haloperidol dose of ≥2 mg in the post-CPOE set group than in the pre-CPOE set group (47.8% vs 64.3%, p<0.048).  Patients in the post-CPOE set group were monitored with ECG more often and were more likely to have an ECG after administered intravenous haloperidol (61.2% for the post-CPOE set group vs 39.3%, p=0.009). In the post-CPOE set group, ECG monitoring 24 hours after a haloperidol dose was conducted more often than in the pre-CPOE set group (58.5% vs 25.2% of the time) | Retrospective study design.  Small sample size. Low external validity.    After implementation, the link did not help in decreasing concomitant use of QT prolonging drugs. | Physicians in an inpatient setting |
| Sorita et al. (2015)^33^  USA | Quasi-experimental study  Mayo Clinic,  Rochester, Minnesota | **Silent phase:**  359 patients  Age (mean, SD): 64.2 ± 18.7  **Active phase:**  648 patients  Age (mean, SD):  63.7 ± 19.1  110 patients belonging to both groups. | Significantly prolonged QTc ≥500 ms | Not specified | Evaluation of efficacy after development and implementation of a CPOE QT alert (clinical decision support) that was triggered when a torsadogenic drug was attempted to be prescribed in patients with documented QT prolongation, found through the QT alert system by Haugaa et al.^21^  In the study there was two phases; the “silent phase” (before implementation of the alert system) and the “active phase”, in which the alert system had been implemented. | Drugs triggering alerts taken from CredibleMeds.^1^  The proportion of completed orders for QT prolonging drugs after an alert were reduced after the system was activated (16.8% [95% CI 14.7-18.9%, p<0.001).  Across all specialties, all provider types and education levels in the clinic, a significant reduction in orders was seen after the activation of the system. | The study could not exclude confounding and dependency of data due to the study design.  Alert fatigue (77% of alerts overridden) | Physicians |
| **Clinical decision support systems (CDSS) (n=6)** | | | | | | | | |
| Bertsche et al. (2010)^20^  Germany | A prospective,  controlled intervention cohort study  was conducted in a medical intensive  and intermediate care unit in a university hospital. | Patients with ≥8 prescribed drugs, based on a pilot study.  **Control group:**  136 patients  Age (mean, SD):  61.0 ± 15.2 years  **Intervention group:** 129 patients  Age (mean, SD)  61.9 ± 14.9 years  Of these, 57 patients remained in the control group and 53 patients remained in the intervention group until day 7 after admission. | QTc interval  >450 ms | Bazett’s correction formula^28^ | Investigation of DDIs and DDI related ADRs^i^ in patients with a developed and pilot tested CDSS containing information on risk and management of 9,453 drug combinations.  CDSS developed by an interdisciplinary team who conducted a systematic search for evidence of drug combinations.  In the control phase only life-threatening DDIs and contraindications from the CDSS were forwarded to a senior clinician.  In the intervention phase, information from the CDSS was approved by a pharmacist and forwarded to a senior clinician, 3 days after patient admission.  ADRs were observed until day 7 after admission, transfer to other units, discharge or death. DDI warnings were only given on day 3. | The incidence of QT prolongation was reduced by 64% from  15 (11%) patients in the control group  to 5 (4%) in the intervention group (p=0.04).  DDIs appeared more frequent in controls (90 patients; 66%) versus the intervention group (70 patients; 54%, p=0.02, RRR^j^: 18%).  The percentage of patients with at least 1 DDI-related ADR was lower in the intervention group (25%) than in the control group (44%) until day 7 after admission (p<0.01, RRR: 43%), mainly due to QTc prolongation and hypokalemia incidence reduction.  Physicians twice as often discontinued a drug after a DDI alert due to the intervention. In the intervention, fewer patients needed a prescription of new medication to treat ADRs (OR: 0.55, p<0.02). | The study was not developed exclusively for investigation of QT prolonging drugs.  Randomization and blinding were not stated, and bias cannot be excluded.  There might have been an underestimation of the occurrence of DDIs and of the prevalence of adverse drugs events in the study. | Pharmacists and physicians in an inpatient setting |
| Tisdale et al. (2014)^36^  USA | Prospective, observational study  Cardiac care units (CCU) at Indiana  University Health Methodist Hospital | **Pre-intervention group:**  1200 patients.  Age: 48% over the age of 67.  **CDSS implementation group:**  1200 patients.  Age: 39% over the age of 67 | QTc interval ≥500 ms or an increase in QTc of ≥60 ms compared with the admitting value at any time during admission. | Bazett’s correction formula^28^ | Investigation of the effectiveness of a CDSS with an incorporated risk score by Tisdale et al.^12^ for reducing the risk of QT prolongation. The CDSS alerted pharmacists entering orders for QT prolonging drugs, who could then discuss risk mitigation strategies with the prescriber.  (1) Pre-intervention: data collection in pre-intervention group, development and validation of the risk score.^12^  (2) Development and modification of the CDSS. Incorporation of the risk score^12^. The CDSS was shown if a QT prolonging drug was ordered, the patient had a moderate or high risk score^12^ or admitting QTc >500 ms. Pharmacy and physician stuff were educated about the system.  (3) Intervention testing: data collection in CDSS implementation group, assessment of the CDSS, impact of the CDSS. | Implementation of the CDSS reduced the risk of QTc prolongation, adjusted OR 0.65 (95% CI 0.56-0.89; p<0.001).  The CDSS reduced prescribing of torsadogenic non-cardiac drugs, adjusted OR, 0.79 (95% confidence interval, 0.63–0.91; p=0.03).  The percentage of patients with a high risk score was lower after the implementation of the CDSS (4.4% vs 10.3%, p<0.001).  Validated risk score (see Tisdale et al.^12^). | Limitation in generalizability (see Tisdale et al.^12^).  Selection bias.  Alert fatigue (82% of alerts overridden). | Pharmacists and physicians in an inpatient setting |
| Böttiger et al. (2017)^19^  Sweden | Development of a CDSS in a pilot study. Survey.  Two geriatric wards, three primary health care centers | **Pilot study:** 503 patients from geriatric wards and 368 primary care patients.  **Surveys:**  Pre-study questionnaire respondents;  32 primary care physicians and  29 geriatricians.  2^nd^ questionnaire after 4 months from starting to use the CDSS: Results are based on responses of 17 primary care physicians and 15 geriatricians who had actually used the CDSS. | - | - | Development of PHARAO (Pharmacological Risk Assessment Online), a CDSS presenting a risk profile for adverse drug events.  1427 substances were scored from 0 (no pharmacological effect) to 3 (strong pharmacological effect) for the following eight properties: risk of bleeding, sedation, orthostatism, constipation, anticholinergic and serotonergic side effects, **QT prolongation/**  **arrhythmia** and seizures. Substances with nephrotoxicity was scored 0-1.  Algorithms were developed for creation of individual risk profiles from medication lists.  A pre-study questionnaire about expectations on PHARAO was sent to physicians. A post-study questionnaire for evaluation of PHARAO was also sent to physicians. | PHARAO was based on pre-clinical and clinical evidence of receptor affinity and clinical evidence of side effects.  Documented experiences by physician.  The mean grading of the usefulness of PHARAO (scale 1-6) was 3.7.  PHARAO was considered easy to use and supported medication review by most physicians.  21/32 physicians would recommend PHARO, additionally 5 physicians would recommend it with modifications. | PHARAO wasn’t developed exclusively for risk assessment of QT prolongation.  PHARAO needs to be evaluated regarding unnecessary generated signals.  2/3 of the respondents to the first questionnaire were specialist physicians. | Physicians |
| Berger et al. 2020^37^  The Netherlands | Prospective, observational study | **Development cohort**: 107 patients, age: 56.0 (median)  **Validation cohort**: 1579 patients, age: 77.0 (median)  A model was developed based on risk factors associated with QTc-prolongation determined in a prospective study on QT-DDIs^b^ .  The ability of the model to predict QTc-prolongation was validated in an independent dataset obtained from a general teaching hospital against QTc-prolongation as measured by an ECG as the gold standard. | QTc interval > 450 ms for males and > 470 ms for females. | Fridericia formula | The model included age, gender, cardiac comorbidities, hypertension, diabetes mellitus, renal function, potassium levels, loop diuretics, and QTc-prolonging drugs as risk factors. Application of the model resulted in an area under the ROC-curve of 0.54 (95% CI 0.51–0.56) when QTc-prolongation was defined as > 450/470 ms, and 0.59 (0.54–0.63) when QTc-prolongation was defined as > 500 ms. A cut-off value of 6 led to a sensitivity of 76.6 and 83.9% and a specificity of 28.5 and 27.5% respectively. | Tool is based on seven predictors, that could easily be implemented in everyday practice.  The model was externally validated using an independent dataset of a general hospital, showing the robustness of the model.  High sensitivity of the model (76.6%-83.9%) identifies patients at high risk for TdP | Quite low specificity (27.5%) means that the model incorrectly labels patients at risk for QTc-prolongation.  Ideally, the model shoud be developed and validated with TdP as primary outcome. That was not possible in this study. | Healthcare providers |
| Chernoby et al. 2020^38^  USA | A multicenter, retrospective quasi-experimental study  Ascension Southeast Michigan, consisting of 5 community teaching hospitals that use a common EMR and drug interaction platform. | Patients with a known risk of TdP with QTc greater than 500 ms  **Silent phase** (for testing purpose of the QT-CDS):  49 patients  Age: 67.3 (15.1)  **Active phase:**  100 patients  Age: 66.2 (15.8)  Implementation of the QT-CDS led  to a reduction in the proportion of QTc alert–generating medication orders continued with no intervention (from 81.6% in the silent phase to 37% in the active phase, an absolute reduction of 54.6%). | QTc greater than 450 ms | Bazett’s correction formula^28^ | The QT-CDS was designed to fire an alert each time a prescriber attempt to order a QTc prolonging drug in a patient with QTc greater than 500 ms. A copy of the most recent ECG report could be displayed right from the alert.  The risk of developing QTc prolongation was calculated using a previously validated scoring system of Tisdale et al.^12^ The following variables were collected: age >68 years, female gender, use of loop diuretic, potassium concentration less than 3.5 mEq/L, admission QTc > 450 ms, acute  MI, use of QTc-prolonging medications, sepsis, and heart failure | The QT-CDS tool decreased by 54.6% the proportion of QTc alert-generating orders continued without intervention. Alerts provided in the tool included additional patient-specific data to facilitate decision making.  The override rate of alerts (37%) was significant lower than rates reported in previous studies) | Retrospective nature  Reliance on accuracy of documentation  Administration of as-needed orders were not collected  The quasi-experimental desing of the study was unable to account for secular trends in prescribing across the study period | Physicians |
| Berger et al. 2021^39^  The Netherlands | Intervention study using a pre- and post-design in 20 community pharmacies in The Netherlands | A total of 928 QT-DDI alerts were generated during the pre- and post-CDS tool phases.  **Before-period:** patients n= 233 (median age 66) and QT-DDI alerts n= 244,  **After-period**: patients n= 149 (median age 63) and QT-DDIs n=157  There was no significant difference in the proportion of QT-DDIs for which an intervention was made after implementing the tool: 43.0% before and 35.7% after implementation (OR 0.74; 95% confidence interval 0.49–1.11). Substitution of interacting agents was the most frequent intervention. | QTc-prolongation  was defined as >500 ms | not mentioned | The CDS tool consisted of a paper-based flowchart identifying patients that were at increased risk for developing QTc-prolongation.  All QT-DDIs that occurred during a pre- and post-CDS tool period of 3 months were included. The QTc-prolonging drugs involved in the QT-DDIs are listed at the CredibleMeds^1^ | The study focused on CDS tool to support the handling of QT-DDIs in community pharmacies.  Pharmacists spend less time on the management of QT-DDIs when the CDS tool was used. | The tool was paper-based and was not integrated into the electronic CDS system of the community pharmacies.  The documentation of the QT-DDIs was limited; only 48 forms were completed versus the 157 QT-DDIs generated by the CDS system.  Laboratory values retrieved from the pharmacy information system was low.  Four pharmacies dropped out of the study due to construction work of pharmacys and shortages of personnel. | Pharmacists |
| **Other risk assessment tools (n=3)** | | | | | | | | |
| Chan et al. (2007)^40^  Australia | Retrospective case-controlled study, in which cases of drug-induced TdP were searched for in a systematic review | **Cases:**  130 cases of drug-induced TdP.  Age (median):  53 years (interquartile range 36–68 years; range 10–95 years)  **Controls:**  318 overdose patients who had used non-cardiotoxic drugs. Age (median):  34 years (interquartile range 23–45  years). | QTc values of 440 ms and  500 ms: medium and high-risk values,  respectively | Did not require heart rate correction, but the risk assessment tool was compared with Bazett’s correction formula^28^ | A QT nomogram, which was derived from a cloud diagram developed for human preclinical studies (Fossa et al.^41^).  Both the QT interval and heart rate of cases and controls were plotted with the QT nomogram. On the nomogram there was an “at risk” line, if the patient’s measurements went above the line, they were at risk of TdP.  Additionally, two curves corresponding to Bazett’s correction formula at QTc values of 440 ms and 500 ms were plotted. Analysis was also made with no extrapolation, all cases with heart rate >104 bpm were excluded from analysis. | **Sensitivity % (95 % CI):** QT nomogram: 96.9% (93.9-99.9),  QT nomogram (no extrapolation): 98.3% (96.1-100).  **Compared to:**  Bazett’s QTc=440 ms and 500 ms, respectively:  98.5% (96.4-100); 93.8% (89.6-98.0).  **Specificity % (95% CI):**  QT nomogram 98.7% (96.8-100),  QT nomogram (no extrapolation): 99.3% (97.8-100).  **Compared to:**  Bazett’s QTc=440 ms and 500 ms, respectively:  66.7% (58.6-74.7); 97.2% (94.3-100) | Retrospective study due to the rarity of TdP cases.  The study design and further description of studies included in the systematic review was lacking. The systematic review did not assess risk of bias.  Risk of publication bias since the positive cases of TdP were extracted from the literature.  The validity of some of the case points at tachycardia were questionable. | Physicians |
| Berling and Isbister (2015)^43^  Australia | Retrospective analysis of prospectively gathered observational data. | Positive controls and negative controls obtained from Chan et al.**^40^**  **Positive controls:** 129 cases of drug-induced TdP as positive controls.  **Negative controls:** 316  QT–heart rate pairs from an overdose dataset of non-  cardiotoxic drugs as negative controls. | Abnormal QT deﬁned as a QT greater than half of the RR interval.  Abnormal QT deﬁned as greater than 500 ms for correction formulae | Did not require heart rate correction.  The risk assessment tool was compared to Bazett’s and Fridericia’s correction formulae^28,29^ | The ½ RR rule deﬁned a QT interval as abnormal if the measured QT was greater than half the RR interval for the same ECG.  The ½ RR rule was compared in the study to the QT nomogram^40^ and Bazett’s and Fridericia’s QT correction formulae^28,29^ | Rapid assessment of the QT interval | Retrospective analysis.  Risk of publication bias since the positive cases of TdP were extracted from the literature.  Poor positive and negative agreement.  **Sensitivity, % (95% CI):**  ½ RR rule:  87.6 (80.4–92.5).  Compared to:  QT nomogram: 96.9 (91.8–99);  QTcB^k^ > 500 ms: 93.8 (89.6–98);  QTcF^l^ > 500 ms: 82.2 (75.6–88.8).  **Specificity, %**  **(95% CI):**  ½ RR rule:  52.9 (47.2–58.4).  Compared to:  QT nomogram:  98.7 (96.6–99.6);  QTcB > 500 ms 97.2 (94.3–100);  QTcF > 500 ms: 100 (100–100). | Health care providers in emergency departments |
| Sugrue et al. (2015)^44^  USA | Retrospective study  Mayo Clinic in Rochester, Minnesota. | **Sotalol cases**, n=8.  Age (mean, SD): 60.3 ± 14.5  **Dofetilide cases,** n=5.  Age (mean, SD): 68.4 ± 5.5  **Sotalol controls**,  n=16.  Age (mean, SD): 61.4 ± 14.0  **Dofetilide controls**, n=10.  Age (mean, SD): 68.0 ± 5.4 | QTc >500 ms associated with a higher risk of lethal arrhythmias. Prolonged QTc for female sex:  460 ms | Bazett’s correction formula^28^ | Identification of electrocardiographic predictors of  torsadogenic risk through the application of a T wave analysis tool.  The software program provided extraction of information from automatic 12-lead ECGs and T wave morphological changes were analyzed.  For risk assessment, in addition to the QTc, T wave parameters have potential to enhance risk stratification. | Heart rate corrected QT interval discriminated TdP cases from controls in 79%.  The discrimination increased to 88%, when adding the T wave right slope in the analysis, | Retrospective analysis.  Small sample size.  Potential of confounding factors.  Requires validation in comprehensive population. | Health care providers |
| ^a^ ECG, Electrocardiogram ^b^ SD, Standard deviation ^c^ HR, Hazard ratio ^d^ CI, Confidence interval ^e^ OR, Odds ratio ^f^ PPV, Positive predictive value ^g^ NPV, Negative predictive value ^h^ DDI, Drug-drug interaction ^i^ ADRs, Adverse drug reactions ^j^ RRR, Relative risk reduction ^k^ QTcB, QT interval correction with Bazett’s correction formula ^l^ QTcF, QT interval correction with Fridericia’s correction formula | | | | | | | | |
